# Field evaluation of the enhanced MM3-COPRO ELISA test for the diagnosis of *Fasciola hepatica* infection in sheep

**DOI:** 10.1371/journal.pone.0265569

**Published:** 2022-03-24

**Authors:** Mercedes Mezo, Marta González-Warleta, José Antonio Castro-Hermida, Victoria Martínez-Sernández, Florencio M. Ubeira

**Affiliations:** 1 Laboratorio de Parasitología, Centro de Investigaciones Agrarias de Mabegondo, AGACAL, Abegondo, A Coruña, España; 2 Laboratorio de Parasitología, Facultad de Farmacia, Universidad de Santiago de Compostela, Santiago de Compostela, España; US Geological Survey, UNITED STATES

## Abstract

Fasciolosis is a severe zoonosis responsible for major economic losses in livestock. The enhanced MM3-COPRO test (eMM3-COPRO) and the commercial version BIO K 201 (Bio-X Diagnostics, Rochefort, Belgium) are widely used as immunodiagnostic tools for the specific detection of coproantigens released by *Fasciola* during the late prepatent and patent stages of infection. However, performance of the eMM3-COPRO has never been evaluated under field conditions. To address this gap, a large number of ovine faecal samples, collected in a region where fasciolosis is endemic (Galicia, NW Spain), were analyzed. Two groups of sheep flocks were selected according to the *Fasciola* infection status: ‘*Fasciola*-free’ and ‘*Fasciola*-infected’ flocks. ‘*Fasciola*-free’ flocks were seronegative flocks with no history of fasciolosis detected by either coproscopy or necropsy in the last 5 years. Faecal samples from these sheep were used to calculate a cut-off value for infection (OD = 0.021). The cut-off was calculated using a bootstrap resampling method that enables estimation of the sampling distribution of the statistical parameters without making assumptions about the underlying data distribution. ‘*Fasciola*-infected’ flocks were characterized by high seroprevalence, a history of fasciolosis and periodical treatment with flukicides. Samples from these flocks were used to estimate the diagnostic accuracy of the eMM3-COPRO relative to coproscopy, which although limited by poor sensitivity is the only reference test available for diagnosing fasciolosis *in vivo*. To overcome this limitation, all animals classified positive by eMM3-COPRO were treated with triclabendazole and then retested. The eMM3-COPRO displayed higher sensitivity than coproscopy, as it detected coproantigens in all samples with positive coproscopy and in 12% of samples with negative coproscopy. The test also proved highly specific as coproantigens disappeared after the treatment. The eMM3-COPRO was less time consuming than coproscopy, particularly when the procedure involved numerous samples, and showed promise as a tool for monitoring flukicide efficacy.

## Introduction

Fasciolosis, caused by *Fasciola hepatica* and *Fasciola gigantica*, is a severe zoonosis that causes major economic losses in pasture-fed ruminant production systems [1–5]. Ruminants, which are reservoirs of the infection, become infected when they accidentally ingest the infective stage of the parasite (metacercariae) along with grass and water. Metacercariae excyst in the small intestine releasing the juvenile flukes, which travel through the duodenal wall to the liver. In liver parenchyma, flukes feed and grow for 6–8 weeks, before finally entering the bile ducts. At this location, they reach sexual maturity, at 8–10 weeks post-infection (pi), and start to lay eggs, which are shed along with faeces.

Ideally, *Fasciola* infections should be diagnosed early, to prevent severe damage to the liver tissue and also environmental contamination with fluke eggs, particularly those from treatment-resistant parasites [6]. Reliable assessment of the efficacy of flukicides is therefore also an essential requirement for the success of any fasciolosis control programme [7].

Immunological techniques, which detect circulating antibodies to *Fasciola* from 1–4 weeks pi, are attractive tools for early diagnosis of fasciolosis [8–11]. However, antibodies can remain detectable for some time after removal of the fluke burden by successful treatment, and therefore these techniques cannot differentiate between current and past infections [12]. This limitation makes them unreliable for diagnosis in endemic areas, where treated animals can become re-infected.

Other diagnostic techniques are based on the detection of material sourced by *Fasciola* (eggs, DNA or metabolic antigens) in host faeces. Identification of fluke eggs by coproscopy is only possible after the beginning of the patent period (i.e. from 8–12 weeks pi), when liver tissue has already been damaged and pastures have been contaminated. Detection of DNA from *Fasciola* by different molecular methods has recently been used for diagnostic purposes [13–15]. The main advantage of molecular techniques is that they enable identification of the species involved in the infection, which is of interest in areas where *F*. *hepatica* and *F*. *gigantica* co-exist [15]. However, these techniques suffer from the same insurmountable limitation as coproscopy, because parasite eggs are the source of the DNA to be amplified.

Some metabolic antigens produced by late immature and adult flukes are released into the bile and passed in faeces before egg laying starts. To detect such coproantigens, several capture ELISA techniques have been developed in the last few decades [16–19]. However, only the in-house MM3-COPRO ELISA and the commercial version BIO K 201 kit (BIO-X Diagnostics, Rochefort, Belgium) have been globally tested [14, 20–26]. Following their widespread use, both tests have been recognized as useful tools for specific diagnosis of early infections in both ruminants and humans [11, 20, 27–32], as well as for monitoring flukicide treatments [6, 28, 29, 31, 33–36]. Nonetheless, some reports have also emerged regarding failures of sensitivity [37–39]. The MM3-COPRO ELISA has therefore recently been modified to enhance its performance, even with short incubations [40]. In this work, we assessed the diagnostic and operational performance of this enhanced version of MM3-COPRO ELISA (eMM3-COPRO) by using a large number of samples collected from different commercial sheep flocks distributed throughout Galicia (NW Spain), where fasciolosis caused by *F*. *hepatica* is endemic.

## Materials and methods

### Ethical approval

This study was carried out in strict accordance with the guidelines of European Directive 2010/63/EU and Spanish Law RD 53/2013 on the Care and Use of Laboratory Animals. The protocol was approved by the Ethics Committee of the Consellería do Medio Rural of the Xunta de Galicia (Spain).

### Flocks

**Fig 1:**
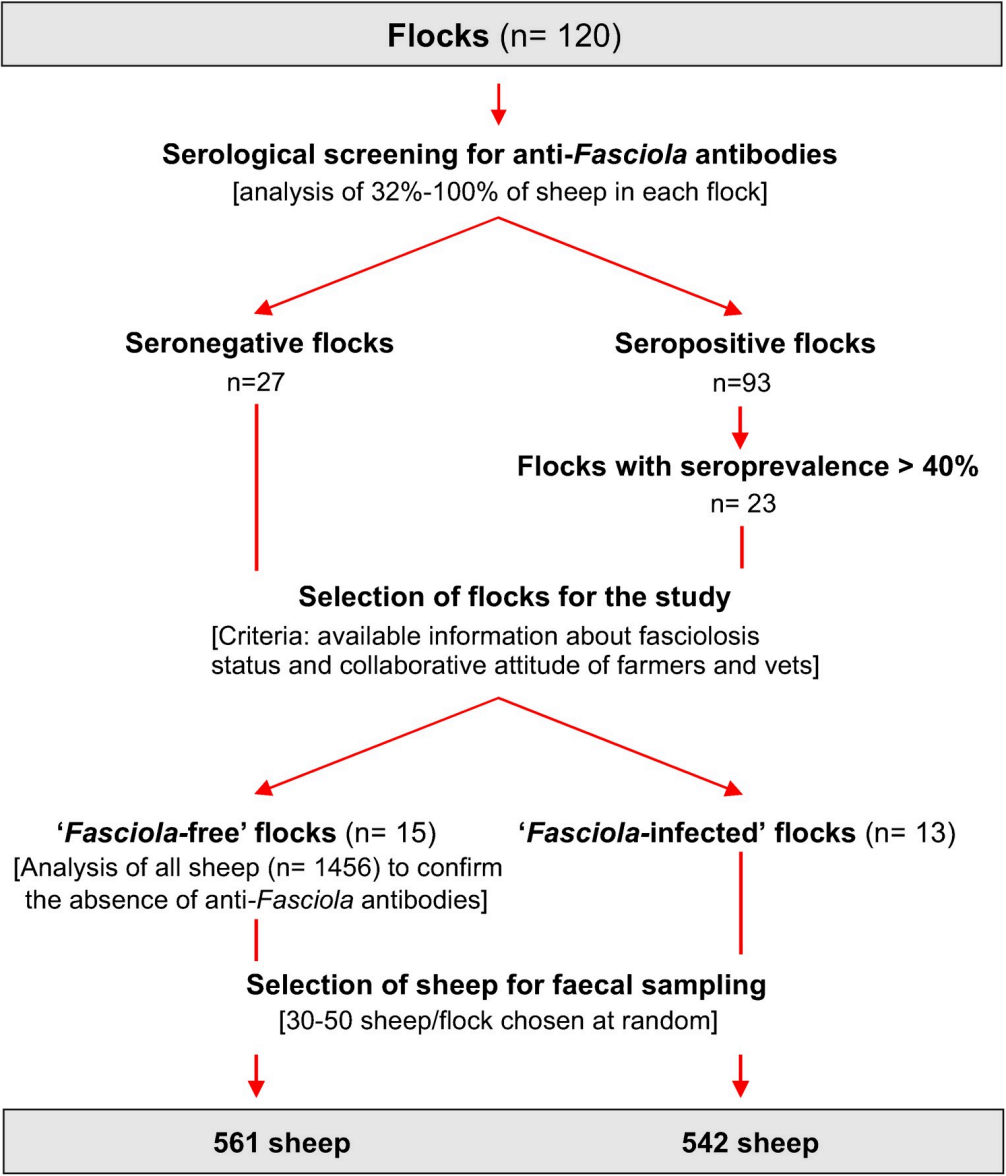
Diagram showing the process of selection of the 1103 sheep tested in the study.

The sheep flocks involved in the study were selected in two phases (Fig 1). First, serological screening for anti-*Fasciola* IgG antibodies was carried out in 120 pastured sheep flocks distributed throughout the region. Serum samples were obtained from 32–100% sheep in each flock, yielding a total of 6950 samples. The samples, provided by the Official Veterinary Services, had been collected for monitoring other diseases included in the regional animal health programme. In this phase, 27 seronegative flocks and 23 flocks with high seroprevalence (>40%) were selected. Secondly, owners of the selected flocks and attending veterinarians were asked for information about previous 5-year history of fasciolosis (records of the routine coproscopies, on-farm necropsies, data provided by slaughterhouses and flukicide treatments). For 15 of the 27 seronegative flocks and 13 of the 23 flocks with high seroprevalence, sufficient information was provided to confirm the status of *Fasciola* infection; the flocks were selected and classified as follows: a) ’*Fasciola*-free’ (seronegative flocks in which liver fluke infection was not detected in either routine coproscopies or necropsies) and b) *’Fasciola*-infected’ (flocks with high seroprevalence, history of fasciolosis and periodical treatment with flukicides). All animals in the ’*Fasciola*-free’ flocks were re-tested to confirm their seronegativity prior to collection of the faecal samples used in this study. All serological analyses were performed with the MM3-SERO ELISA test, as described by [8].

### Faecal samples

**Fig 2:**
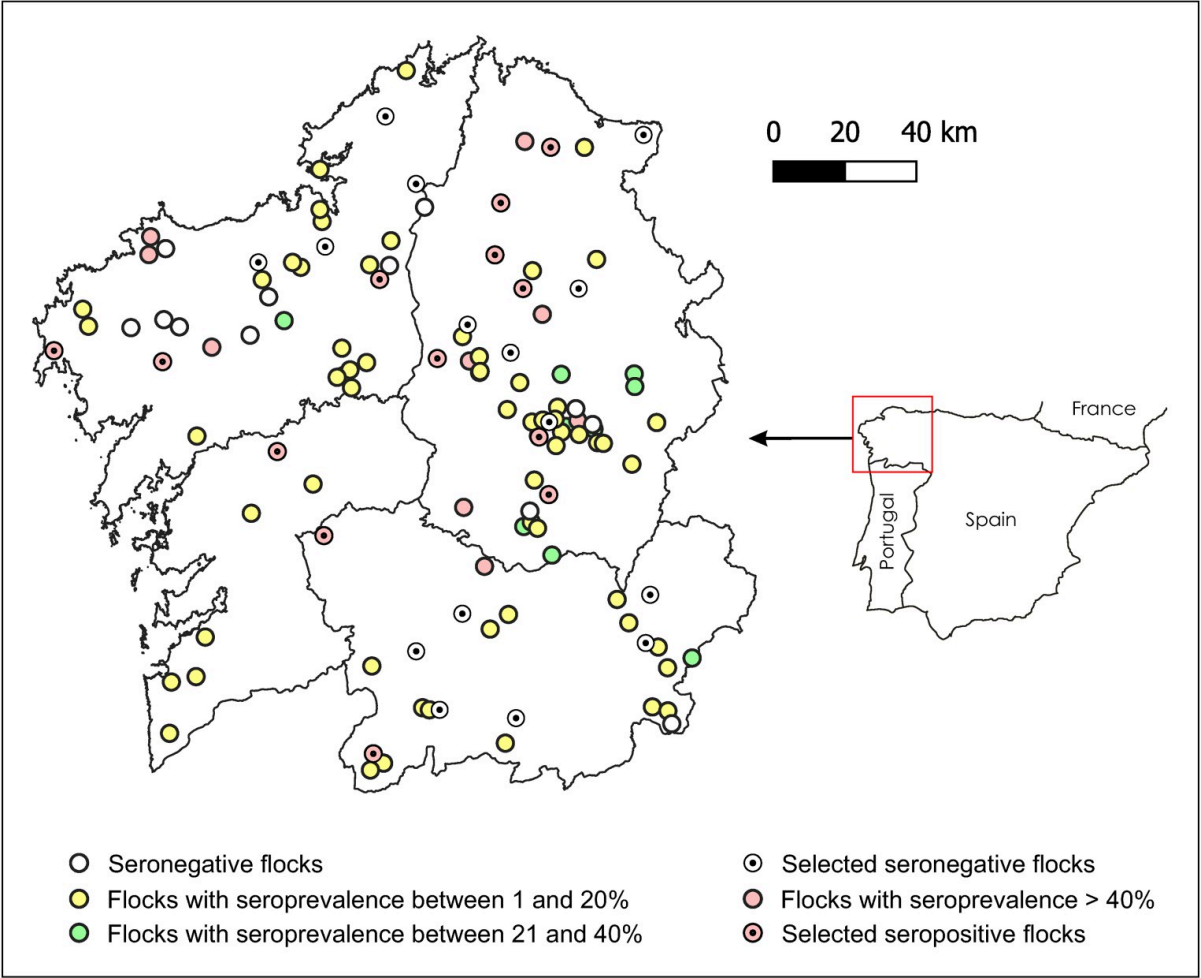
Map of Galicia. The location of the starting 120 sheep flocks categorized according to their anti-*Fasciola* antibody status is shown. The flocks finally selected for the study are also displayed. Reprinted from BDLJE under a CC BY license, with permission from ign.es, original copyright 2015.

As indicated in Fig 1, a total of 1103 sheep (561 from ‘*Fasciola*-free’ flocks and 542 from ‘*Fasciola*-infected’ flocks) were tested in the present study. To ensure that sampled sheep could have become infected by *Fasciola* or other helminths, only those sheep meeting the following 3 criteria were selected: age over 6 months, a grazing period of more than 3 months, and absence of flukicide treatments within the last 6 months. The samples were derived from flocks covering the entire region (Galicia, NW Spain) (Fig 2).

Sampling was conducted between 3 October, 2018 and 15 January, 2019. Samples were collected from the rectum into plastic gloves and then refrigerated (4°C-8°C) until analysis by both coproscopy (within 5 days of sampling) and the eMM3-COPRO test (analysis within 2 days of sampling). As all samples were processed by various techniques (see below), the total number of analyses performed was very high, and replicate analyses were therefore not possible. All analyses were carried out in the parasitology laboratory at the Centro de Investigaciones Agrarias de Mabegondo.

The samples obtained from *’Fasciola*-free’ flocks (n = 561) were used to establish the cut-off value of the eMM3-COPRO, while the samples collected from the ’*Fasciola*-infected’ flocks (n = 542) were used to assess the analytical and diagnostic performance of the test. For evaluation of the performance of eMM3-COPRO, we used the results obtained by coproscopy as reference values, as there are currently no other methods available for the diagnosis of active fasciolosis *in vivo*. However, the sensitivity of coproscopy is known to be low [41–45], which is a huge disadvantage regarding the diagnostic evaluation of more sensitive techniques [46]. Consequently, animals that test positive for coproantigens and negative for fluke eggs may be true positives. To overcome this drawback, all animals that tested positive for coproantigens were treated with triclabendazole (TCBZ) (10 mg/kg; Endex^®^ 8.75%, Elanco Valquímica S.A., Madrid, Spain), and the faeces were retested with the eMM3-COPRO test, 21 days post-treatment.

### Coproscopy

The selected faecal samples (n = 1103) were examined under a microscope (at 40x or 100x magnification) and *Fasciola* eggs were counted. In addition, the samples from *’Fasciola*-free’ flocks (n = 561) were also examined to detect eggs, larvae or oocysts of other parasites with high prevalence in the region. Prior to microscopic examination, faeces were processed by the traditional techniques of sedimentation, flotation and migration.

Trematode eggs were concentrated by a simple sedimentation technique [41]. For all faecal samples, a 5 g aliquot was mixed with about 100 ml of tap water, and glass beads were added to help thoroughly break down the faecal matter. The suspension thus obtained was filtered through a 150 μm sieve before being transferred to a conical flask, diluted to 500 mL with tap water and allowed to settle (3 times, each for 20 min). The final sediment was re-suspended in 5 ml of water, and a 1 ml subsample was used for the microscopic examination. The sensitivity reached was 1 egg per gram of faeces (EPG). Analysis was quantitative for *Fasciola* eggs, but only qualitative for eggs of other trematodes.

Nematode and cestode eggs and coccidian oocysts were concentrated by flotation (Improved Modified McMaster method described by [47]). The faecal samples were mixed with water at a ratio 1:15 (3 g of faeces + 42 ml of tap water) and filtered through a 150 μm sieve. Aliquots (15 mL) of the filtrate were centrifuged for 2 min at 1500 rpm. The sediment obtained was re-suspended with saturated NaCl solution (specific gravity 1.19), and the resulting suspension was charged into a standard McMaster chamber. Both grid areas in the chamber (0.3 ml) were examined under a microscope, yielding a sensitivity of 50 eggs/oocysts per gram of faeces.

A routine migration test [48] was used to collect active lung nematode larvae. Specifically, a 5 g portion from each faecal sample was enclosed in surgical gauze and placed in a Baermann funnel filled with water at room temperature. After 24 h, about 10 ml of the fluid was drawn off from the bottom of the funnel to a tube, which was left to rest at 6-8°C for 2 h. The whole sediment was examined under a microscope.

### Analytical procedures with the eMM3-COPRO test

#### eMM3-COPRO ELISA determinations

ELISA plates were prepared as previously described by [49]. Briefly, polystyrene microtiter 96 well 1x8 strip plates (Greiner Bio-One; Soria-Melguizo, Madrid, Spain) were coated overnight with 100 μL/well of a solution containing rabbit anti-*Fasciola* polyclonal IgG antibodies (wells in the odd-numbered rows) or IgG antibodies from non-immunized rabbits (wells in the even-numbered rows), both at a concentration of 10 μg/ml in phosphate-buffered saline (PBS; 10 mM sodium phosphate buffer, 150 mM NaCl, pH 7.4). Plates were washed three times with PBS and then blocked with 1.5% sodium caseinate in PBS for 1 h at room temperature.

Analysis was performed as described by [40], although with a slight modification in the sample processing procedure, which consisted of replacing the distilled water with CoproGuard (Inmunogal SL, Santiago de Compostela, Spain), a preservative that contains biocidal substances, proteins and surfactants [49]. The samples were mixed with CoproGuard at a ratio 1:4 (1 g + 4 ml), and the suspension was centrifuged for 15 min at 1,000 g to yield the supernatant. Positive and negative control samples were also prepared. The supernatants from 20 negative faeces were mixed, and then split into two parts, one of which was spiked with *F*. *hepatica* excretory-secretory antigens at a concentration of 5 ng/ml. Both portions were aliquoted and stored at -20°C until being used as positive and negative controls in each plate.

The test included the following steps:

1. Addition (100 μL /well) of each supernatant in duplicate (1 odd-numbered well plus 1 even-numbered well) and incubation (room temperature, 30 min) with shaking (750 rpm).
2. Addition (100 μL/well) of biotinylated monoclonal antibody MM3 (diluted 1:10,000) and incubation (room temperature, 30 min) with shaking (750 rpm).
3. Addition (100 μL/well) of streptavidin-polymerized HRP conjugate diluted 1:8,000 (Pierce, Thermo Fisher Scientific, Madrid, Spain) and incubation (room temperature, 30 min) with shaking (750 rpm).
4. Addition (100 μL/well) of the TMB substrate (TMB ONE™ ELISA HRP Substrate, Kementec Solutions A/S, Tasstrup, Denmark) and incubation (room temperature, 20 min) in darkness.
5. Addition (100 μL/well) of 0.2M H_2_SO_4_ and measurement of the optical density (OD) at 450 nm.

After steps 1, 2 and 3 were completed, the plates were washed 6 times with PBS containing 0.05% Tween 20 (PBS-T). All dilutions were made in PBS-T containing 1% BSA (fraction V; Merk Life Science SLU, Madrid, Spain). The plates were shaken on a horizontal orbital shaker with an orbit diameter of 1.5 mm (Microtitre plate shaker SSM5; Stuart Equipment, Staffordshire, UK). The plates were washed with an automated 96-channel microplate washer (Agilent BioTek 405 TS Microplate Washer; BioTek instruments, Winooski, VT, USA). The OD was measured with a spectrophotometer (Tecan Spectra Rainbow A-5082; Tecan Ibérica Instrumentación SL, Barcelona, Spain).

The OD value for each sample was calculated as OD1-OD2, where OD1 is the value for the odd-numbered wells (coated with anti-*Fasciola* antibodies) and OD2 is the value for the even-numbered wells (coated with irrelevant antibodies). Negative values were recorded as zero. OD readings obtained for the negative and positive control samples included in every plate ranged from 0 to 0.005 (mean = 0±0.001) and from 0.828 to 1.145 (mean = 0.969±0.081) respectively.

#### Precision of the eMM3-COPRO test

To determine the precision (also referred to as imprecision) of the assay, 11 positive faecal supernatants with OD values spanning the entire linear range of the assay [40] were repeatedly analyzed. Ten of these supernatants were obtained from faeces from sheep naturally infected by *F*. *hepatica* (as confirmed by coproscopy). Another sample was prepared by mixing the supernatant obtained from a pool of 8 negative faeces with the *F*. *hepatica* excretory/secretory antigen at a concentration of 150 pg/ml, which is the previously reported detection limit of the assay [40]. Each faecal supernatant was divided into 25 aliquots, which were assayed in 5 runs performed on 5 consecutive days (5 replicates per run).

### Statistics

The OD values corresponding to the *’Fasciola*-free’ animals (= reference population) were analyzed by the Kolmogorov-Smirnov test, which showed that the values were not normally distributed, even after log-transformation. Consequently, to estimate the OD value corresponding to the 99th percentile of the population and the 99% confidence interval (CI), we used a bootstrap resampling method [50] that enables the sampling distribution of the statistical parameters to be estimated without making assumptions about the underlying data distribution. In this study, 1000 resamples (without replacement) of size 101 were generated, each with its corresponding 99th percentile statistical value.

The precision of the assay was estimated for different OD values (starting at the detection limit), as recommended by the Clinical and Laboratory Standards Institute (CLSI) [51–53]. The OD results obtained for the 11 repeatedly tested samples were subjected to one-way ANOVA analysis. This enabled determination of both the total variance (which encompassed within and between run variance) and the coefficient of variation (CV) for each sample. The results were expressed as percent CV.

The cut-off value for eMM3-COPRO was established on the basis of the upper limit (UL) of the 99% CI for the 99th percentile. Furthermore, the imprecision was determined as the CV of the assay at OD values close to the detection limit, as recommended by International Office of Epizootics (OIE) for validation of antigen detection assays [54]. Thus, the cut-off value was calculated as *Cut−off* = *UL*+z*UL*CV/100, where UL = upper limit of the 99% CI for the 99th percentile, z = 2.33 (score value from the standard normal distribution for the 99% one-tailed confidence level), and CV = coefficient of variation obtained with the positive sample with the lowest OD values (close to the detection limit). Samples with OD values above the cut-off were considered positive (at a probability level of *P* = 0.01).

The possible association between the OD values obtained with eMM3-COPRO and the faecal egg count (FEC) was evaluated by the Spearman’s rank correlation.

The Kolmogorov-Smirnov test, one-way ANOVA and Spearman’s rank correlation were performed using IBM SPSS Statistics software (version 25.0). Bootstrapping was conducted using R-software (version 4.1.1).

## Results

### The eMM3-COPRO test in the ’*Fasciola*-free’ sheep population

**Fig 3:**
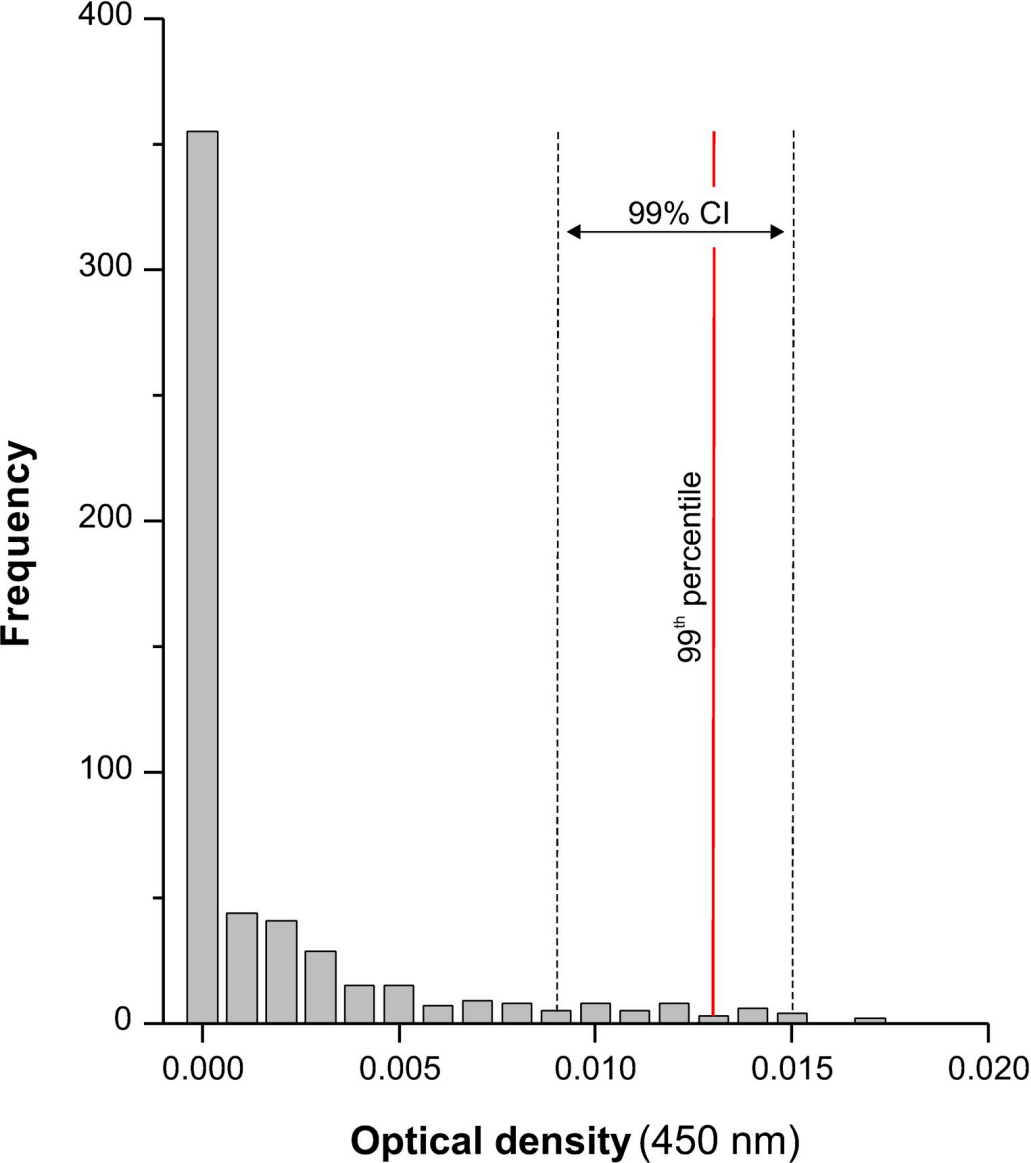
Distribution of OD values obtained with eMM3-COPRO test in the ‘*Fasciola*-free’ sheep population (reference population). The solid vertical red line indicates the 99th percentile value (OD = 0.013) and dotted vertical lines indicate the lower (OD = 0.009) and upper (OD = 0.015) limits of the confidence interval for that percentile (*P* = 0.01). The reference population included 561 sheep from 15 flocks.

The OD values for the 561 samples from the negative reference population were very low (range: 0–0.017) and the data distribution was highly skewed (see Fig 3), with median and 75th percentile values of 0 and 0.002, respectively. The 99th percentile was 0.013 (99%CI: 0.009–0.015).

The OD values were extremely low, despite the fact that coproscopy indicated that all sheep harboured several of the helminths or protozoa included in Table 1. This clearly demonstrates the absence of cross-reactions with the most frequent parasites in the geographical area.

**Table 1:** Helminths and protozoa identified in the population of ‘*Fasciola*-free’ sheep.

**Nematodes**	*Cystocaulus ocreatus* (3.1%) *Muellerius capillaris* (49.1%) *Protostrongylus* spp. (2.6%) *Nematodirus* spp. (8.5%) *Trichuris* spp. (4.3%) Other strongylids, unidentified genus (99.4%)
**Trematodes**	Paramphistomidae, likely *Calicophoron* spp. (12.2%) *Dicrocoelium* spp. (7%)
**Cestodes**	*Moniezia* spp. (17.7%)
**Protozoa**	*Eimeria* spp. (25.7%) *Balantidium*-like ciliates (9.4%)

The percentage of infected animals with each parasite is indicated in brackets.

### Precision profile and cut-off value of the assay

As expected, the precision of the eMM3-COPRO varied depending on the intensity of the ELISA signal (see Table 2). Thus, the CV of the assay was <10% for OD values ≥ 0.162 and between 16.4% and 12.8% for samples with very low OD values (between 0.021 and 0.123).

**Table 2:** Precision profile of the eMM3-COPRO test.

Sample ID^1^	eMM3-COPRO (OD)			
	Range	Mean	SD	CV%
1	0.021–0.038	0.030	0.005	16.4
2	0.023–0.041	0.036	0.005	14.1
3	0.037–0.057	0.046	0.006	13.3
4	0.035–0.058	0.048	0.008	15.7
5	0.062–0.098	0.080	0.011	13.8
6	0.082–0.123	0.104	0.013	12.8
7	0.162–0.221	0.196	0.017	8.6
8	0.342–0.502	0.422	0.042	9.9
9	0.689–0.934	0.799	0.062	7.8
10	1.138–1.507	1.324	0.085	6.4
11	1.478–1.822	1.656	0.082	5.0

^1^Eleven faecal samples with a wide OD range (0.021–1.822) were used to evaluate the precision. Ten samples (ID: 1 and 3–11) were obtained from 10 sheep with natural fasciolosis confirmed by coproscopy, while one sample (ID: 2) was prepared by mixing the supernatant of a pool of 8 negative faeces with *F*. *hepatica* excretory-secretory antigens at a concentration of 150 pg/ml (detection limit of the assay). Each sample was analyzed in 25 independent replicates (5 replicates per run and 5 runs over 5 days). The results obtained were analyzed by one-way ANOVA in order to estimate the total variance (sum of the within and between run variances) and to then calculate both the standard deviation (SD) and the coefficient of variation (CV = SD/Mean) for each sample.

The cut-off value for the eMM3-COPRO was established on the basis of two parameters: the upper limit of the 99% confidence interval for the 99th percentile of the reference population (0.015; Fig 3) and the maximum imprecision of the assay (16.4%; Table 2). Using the formula given in the ’Material and methods’ section, a cut-off of 0.021 was obtained.

### Diagnostic performance of the eMM3-COPRO test. Comparison with coproscopy

The diagnostic accuracy of the eMM3-COPRO (for a cut-off value of 0.021) was assessed on the faecal samples (n = 542) collected from the infected flocks, and the results were compared with those obtained by coproscopy (Table 3). The 221 samples that tested positive by coproscopy were also positive by eMM3-COPRO. However, the results were not concordant for all 321 samples that tested negative by coproscopy, as 39/321 (12.1%) tested positive in the eMM3-COPRO test.

**Table 3:** Results obtained by analyzing the faecal samples collected in the ‘*Fasciola*-infected’ flocks.

eMM3-COPRO	Coproscopy	N	Coproantigen level (OD)		Eggs per gram of faeces	
			Median	Min-Max	Median	Min-Max
+	+	221	0.749	0.029–2.868	12	1–380
+	-	39	0.061	0.022–0.527	-	-
-	-	282	0	0–0.021	-	-
-	+	0	-	-	-	-

Each sample (n = 542) was simultaneously analyzed by the eMM3-COPRO test and coproscopy. In the eMM3-COPRO test, a sample was considered positive when it yielded an optical density value (OD) above the cut-off (OD = 0.021). Coproscopy included counting *Fasciola* eggs (with a sensitivity of 1 egg per gram of faeces).

**Fig 4:**
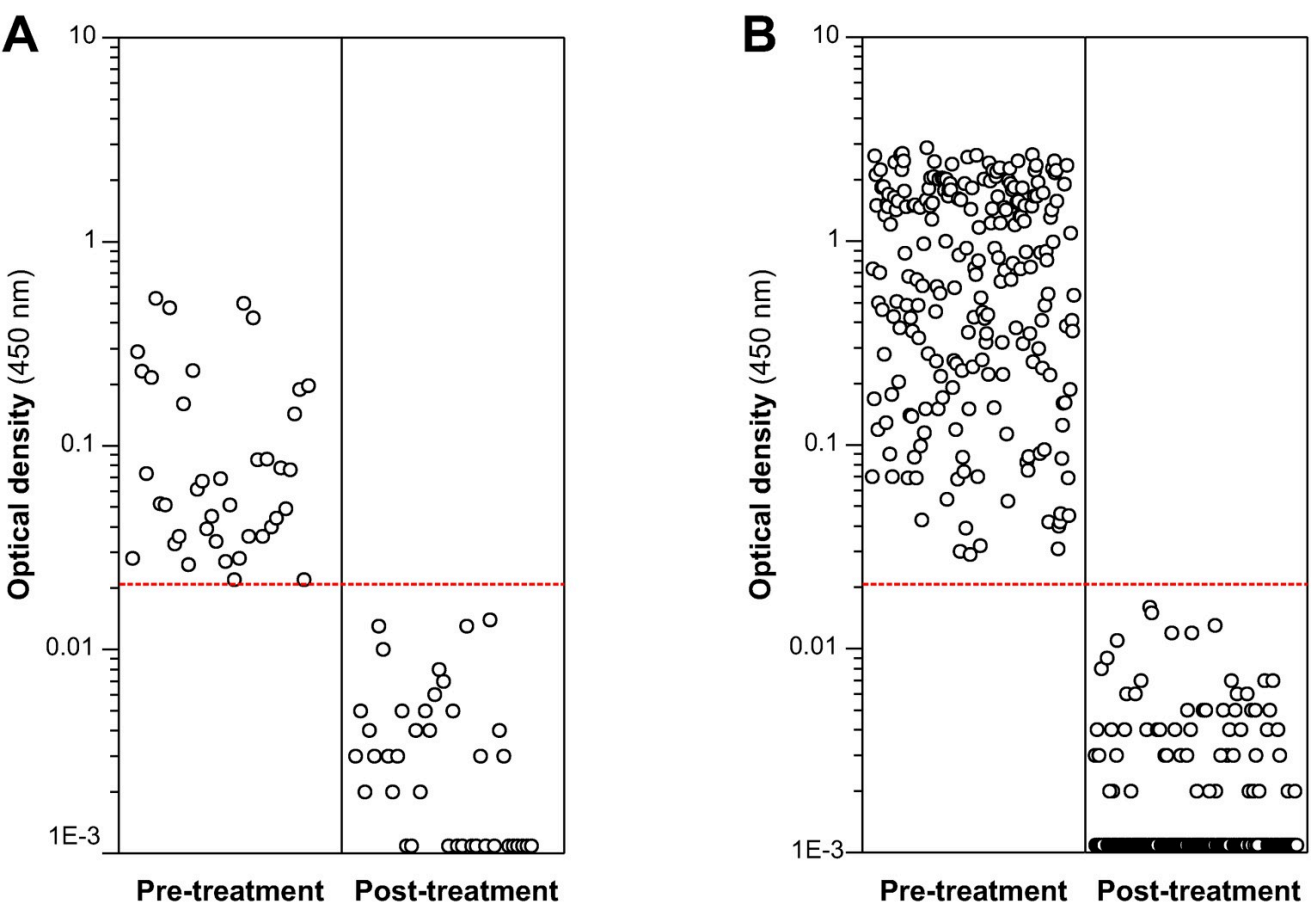
Effect of treatment with triclabendazole on the OD values of sheep testing positive by eMM3-COPRO test. The dose of triclabendazole was 10 mg/kg. Analyses were performed immediately before drug administration and 3 weeks post-treatment. Results are shown separately for sheep with negative (A) and positive (B) coprology. Dashed red line indicates the cut-off value (0.021).

With the aim of elucidating whether this discrepancy reflected either the non-specificity of the ELISA test or a higher sensitivity than coproscopy, the 39 sheep from which these discrepant samples came from were treated with TCBZ and tested again 3 weeks after treatment. As shown in Fig 4A, all OD values decreased to below the cut-off value after treatment, demonstrating that the ELISA test was detecting true liver fluke infections, which were cleared by the treatment. Likewise, the remaining 221 sheep samples that tested positive by both tests (Table 3) also tested negative in the eMM3-COPRO test carried out 3 weeks after the treatment (Fig 4B). These results indicated that the eMM3-COPRO test is highly specific and shows good potential for assessing flukicide efficacy.

**Fig 5:**
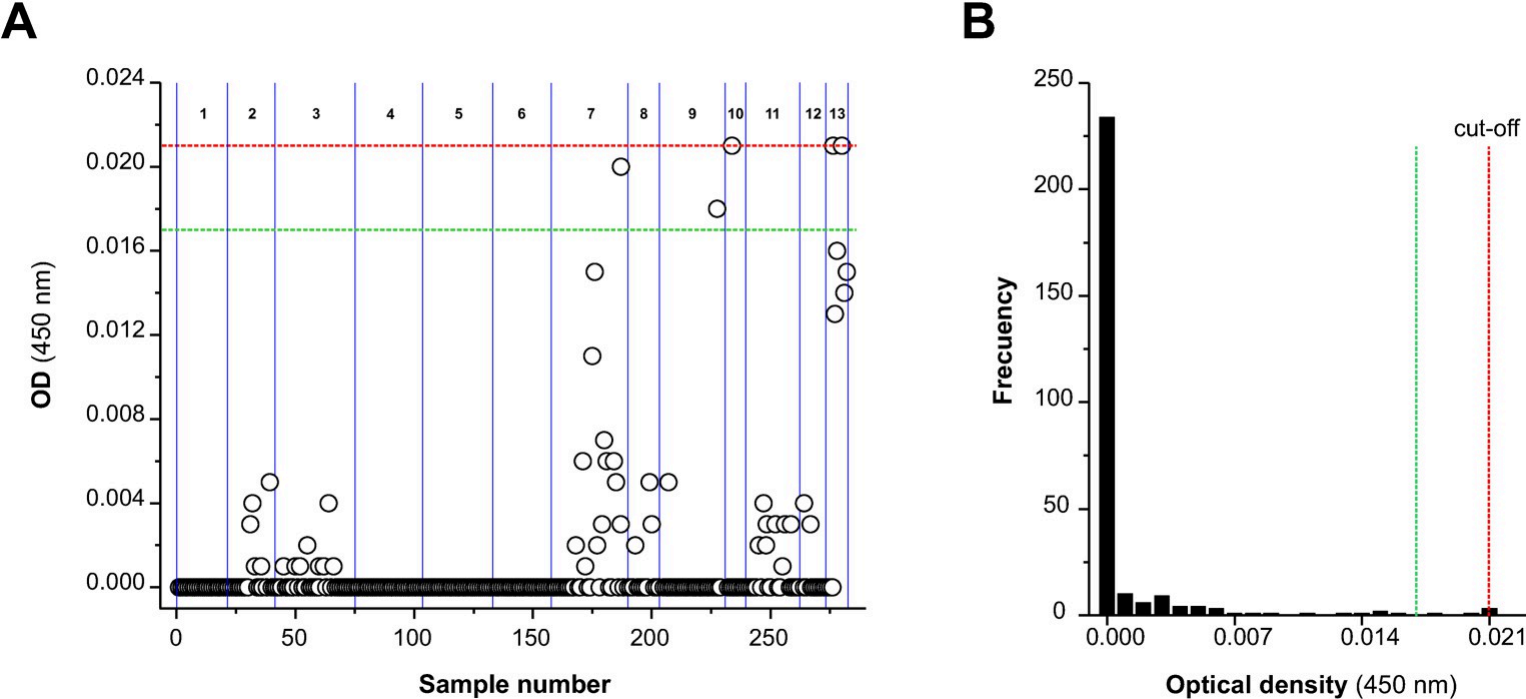
OD values of the ‘*Fasciola*-infected’ flocks’ sheep that tested negative in both eMM3-COPRO and coproscopy. (A) Scatter plot showing the distribution of individual values and (B) diagram of frequencies (n = 282). Dashed red lines indicate the cut-off value (0.021). Dashed green lines indicate the maximum OD value (0.017) in the negative reference population. Solid blue lines indicate the separation between samples from different flocks (1–13).

The OD values of faecal samples from the 282 sheep with a negative result in both the eMM3-COPRO test and by coproscopy ranged between 0 and 0.021 (Table 3). As shown in Fig 5A and 5B, most OD values were much lower than the cut-off value. Indeed, for samples from most sheep (277/282; 98.2%) the OD values ranged between 0 and 0.017, i.e. the range of variation was the same as for the negative reference population. Only samples from 5 sheep (1.8%) from 4 flocks (numbers 7, 9, 10, 13) yielded OD values around the cut-off (between 0.018 and 0.021).

**Fig 6:**
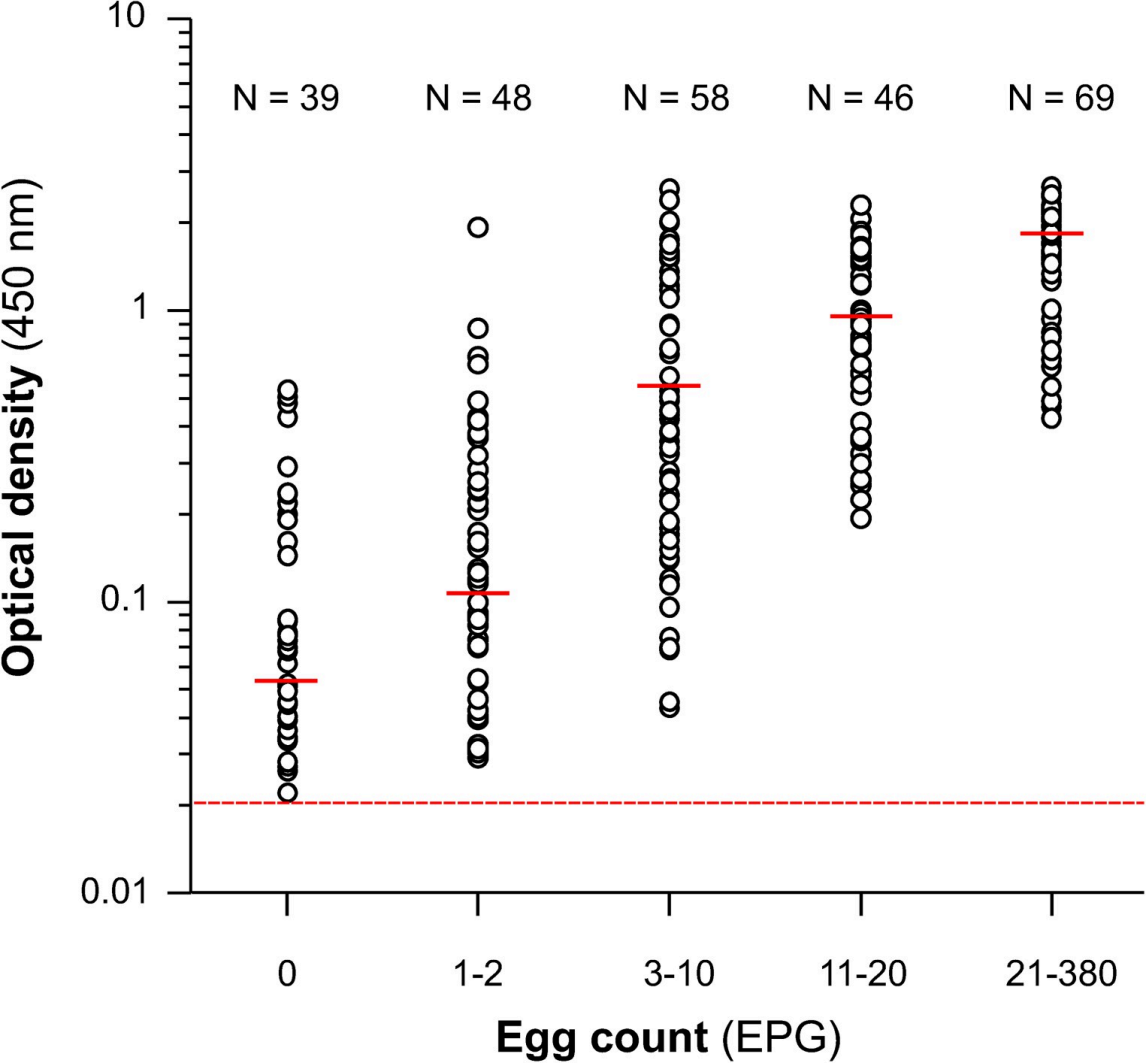
Individual optical density values for the sheep diagnosed as positive by eMM3-COPRO test. Data (n = 260) were clustered into 5 categories based on the faecal egg count. For each category, the median values are represented by a solid horizontal red line. The dashed red line indicates the cut-off value (0.021).

The individual OD values of the 260 ELISA-positive samples, clustered into 5 categories based on the FECs, are shown in Fig 6. As can be observed, coproscopy not only failed to detect eggs in 15% sheep (39/260), but also provided very low FEC (1–2 EPG) for 21.7% of animals with positive coproscopy (48/221). Nevertheless, for sheep that tested positive by both techniques, FECs and OD were significant positively correlated (r = 0.77; *P*<0.001).

### Comparison of the operational characteristics of eMM3-COPRO relative to coproscopy

**Fig 7:**
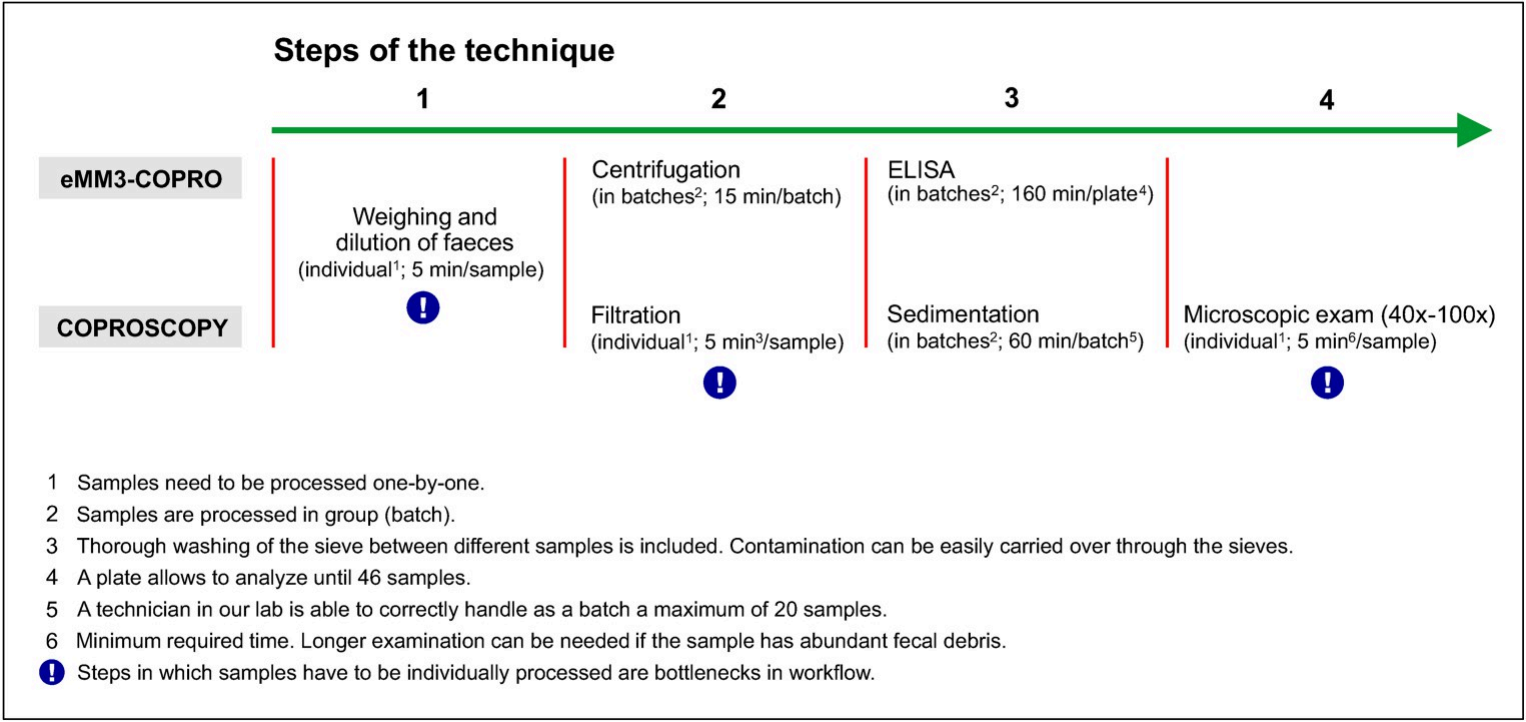
Operational characteristics of eMM3-COPRO test versus coproscopy.

The steps required for processing and analyzing the faecal samples by eMM3-COPRO and coproscopy are shown in Fig 7.

Two key aspects that condition the workflow are indicated for each step: 1) whether samples are processed individually or in batches and 2) the time needed by expert technicians of our laboratory to perform each step. Coproscopy includes three pre-analytical steps (1–3), two of which require samples to be individually processed (steps 1 and 2), while ELISA only has two pre-analytical steps (1–2), and only one of these requires individual processing of the samples. In the analytical step, samples are individually examined in the case of coproscopy (step 4), while batch analysis is conducted in eMM3-COPRO (step 3).

The data shown in Fig 7 enable estimation of the total turnaround times (i.e. the sum of the time taken for each step) for both techniques. The number of samples to be processed and the human and material resources available in each laboratory determine the time required to complete each step, and they must therefore be included in the estimation.

## Discussion

Immunological assays that target *Fasciola* cathepsins are important tools for diagnosing fasciolosis in animals and humans [11, 20, 55]. The detection limit of the eMM3-COPRO test, established in a previous study [40], is 4 times lower than that of the classic version [20], even for shorter incubation times (30 min/step). However, the diagnostic performance under field conditions had not been assessed until now.

In this study, a rigorous cut-off value for the eMM3-COPRO test was established on the basis of the OD values obtained by analysis of a reference population, which met the requirements established by the CLSI regarding both the size (≥120 individuals) and composition (individuals representative of the target population) [56–59]. Specifically, our reference population consisted of 561 *Fasciola*-free sheep chosen at random from different pastured flocks scattered across a region in Spain covering an area of 29,575 km^2^. Moreover, the animals included were raised under the feeding, health and management systems typically used in the region, so that all animals were infected with other endoparasites endemic to the area.

As indicated above, the cut-off value (i.e. the upper limit of the CI of the 99th percentile of the reference population) was calculated using a bootstrap method, a resampling technique that provides accurate estimations of the statistic parameters in the population [60]. The rationale behind establishing this cut-off value is that the CI is a measurement of the uncertainty due to sampling variability, and it should be precisely calculated to estimate the cut-off value for the overall population [57, 61]. The imprecision of a test is another significant factor that affects the reference values [62, 63], and it should therefore also be taken into account to calculate the diagnostic cut-off value. In this case, the values were calculated using the highest CV estimated for the test (16.4%; see imprecision profile), as recommended in the OIE terrestrial manual for development and optimization of antigen detection assays [54]. As the imprecision decreased with increasing OD values, very good analytical performance (CV<10%) was reached for OD values ≥0.162.

The use of a well characterized representative reference population and the application of a standardized statistical approach guarantee that the estimated cut-off is the most appropriate for the target population. The low cut-off value (0.021) obtained in this study is consistent with the high specificity of the MM3-COPRO system for capture and detection of *Fasciola* cathepsins [20, 25, 38, 64, 65]. However, in the original study involving development of the eMM3-COPRO [40], some *Fasciola*-free sheep rendered OD values above 0.021 (up to 0.050). We hypothesize that such differences may be due to the use of different storage conditions and sample diluents in both studies: long storage at -20°C and dilution with distilled water in the former and fresh samples diluted in the CoproGuard preservative in this study. CoproGuard contains tensoactive agents which improve antigen extraction [49] and probably prevent non-specific binding, thus reducing the background signal.

The method used in this study to calculate the cut-off values was also different from that we used during development of the eMM3-COPRO test [40]. Due to the small number of negative samples analyzed (20 samples from sheep and 30 from cattle), the cut-off value in the study was determined as 1 SD above the highest OD value observed on testing the negative samples. Surprisingly, this arbitrary method of calculating the cut-off seems to be adequate, as when applied to the negative samples obtained in the present study (maximum OD value = 0.017 and SD = 0.003) a cut-off value of 0.020 was obtained, which is very close to that obtained with the standardized method (0.021). Receiver Operating Characteristic (ROC) curve analysis, a useful, simple and popular method used to assess the diagnostic accuracy of a test [66], could not be used to calculate the cut-off value of eMM3-COPRO as there is no gold standard method for diagnosing live positive animals.

The sensitivity of the eMM3-COPRO test proved promising, as it detected coproantigens in all samples with a positive coproscopy plus 12% of samples with negative coproscopy. Furthermore, the fact that coproantigens disappeared after treatment with TCBZ (which kills liver flukes, but not other gastrointestinal helminths) is in accordance with the high specificity that this ELISA test displayed for the samples from the ’*Fasciola*-free’ flocks. Previous studies in ruminants with experimental fasciolosis have demonstrated that the release of *Fasciola* coproantigens takes places about two weeks before egg shedding [11, 20, 28, 32, 34]. Therefore, the presence of coproantigens in samples with negative coproscopy may indicate infection with immature flukes (between 5 to 10 weeks of age) or it may be due to very low parasite burdens, which are often misdiagnosed by coproscopy when only one sample is examined [9, 21, 35, 41, 67]. Apart from its activity against liver flukes, TCBZ was recently reported to have some inhibitory activity on bacteria present in gut microbiota [68, 69]. However, this activity is probably not related to the negativization of coproantigens in treated animals for at least three reasons: i) the animals were treated with a single oral dose of TCBZ, and it is therefore unlikely that the bacteria involved were completely cleared without any regrowth within the next 21 days; ii) the OD values obtained in the ‘*Fasciola*-free’ reference population (with gut microbiota similar to that of the treated sheep) were extremely low; and iii) the sandwich ELISA design of eMM3-COPRO containing a polyclonal/monoclonal antibody pair is highly selective for *Fasciola* cathepsins.

Examination of the results for all sheep from ’*Fasciola*-infected’ flocks revealed that most OD values were far enough from the cut-off value that there was no doubt regarding the diagnostic classification. OD values close to the cut-off value (0.018–0.022) were only obtained for 7/542 sheep, 2 with a positive eMM3-COPRO and negative coproscopy result (Fig 4A) and the other 5 with a negative eMM3-COPRO and negative coproscopy result (Fig 5A and 5B). In the 2 sheep with the positive eMM3-COPRO/negative coproscopy result, the OD value decreased from 0.022 to 0 after treatment with TCBZ, and the classification according to the eMM3-COPRO test was therefore probably correct. In the 5 sheep that tested negative by both techniques, no TCBZ treatment was administered. Nevertheless, the fact that the OD values (0.018–0.021) were higher than those corresponding to the 561 sheep in the reference population (0–0.017; Fig 3) and to the 260 treated sheep (0–0.016; Fig 4A and 4B) casts some doubt on the reliability of the classification. The presence of incipient or light infections in these sheep cannot be ruled out.

Detection of prepatent infections is essential so that control programmes can be implemented with the ultimate goal of preventing pasture contamination with *Fasciola* eggs. These programmes often include flukicide treatment, which should be selectively administered and then monitored for efficacy [7]. The method used for assessing drug efficacy has historically been the faecal egg count reduction test (FECRT), which obviously cannot be used in the case of non-patent infections. Furthermore, this test is not very accurate for small fluke burdens and low FECs [12]. In this regard, 33.5% (87/260) of samples that tested positive in this study by eMM3-COPRO had FECs between 0–2 EPG, and monitoring treatment by FECRT would therefore be either impossible or unreliable [70–72]. In such cases, the eMM3-COPRO test may be a useful alternative to FECRT, as all OD values were clearly negative after the treatment. This possibility requires further investigation.

A key point regarding reducing the spread of *Fasciola* is the identification of those sheep that contribute most to pasture contamination. We observed a significant correlation (r = 0.77) between the FEC and the OD signal in the eMM3-COPRO test. This observation is consistent with a previous report of a similar correlation (r = 0.67–0.87) between the FEC and the OD value obtained with the commercial Bio K 201 test in experimentally infected sheep [36]. These results suggest that sheep with higher levels of coproantigen are the main spreaders of infection in the flock.

Regarding the operational characteristics, the eMM3-COPRO test showed the following advantages relative to coproscopy: 1) fewer pre-analytical steps are required, which translates into time saving and lower risk of mistakes; 2) fewer steps requiring individual sample processing are required. Steps demanding exclusive dedication cause bottlenecks, so that removing them improves workflow; 3) it does not include a filtration step, which is associated with a risk of carry over contamination; and 4) a smaller amount of sample is required.

Coproscopic methods used in the diagnosis laboratories can differ significantly in regard to filtration and sedimentation steps. In some methods [31, 42, 43, 73], samples are filtered through a stack of sieves of decreasing mesh opening, so that faecal debris is retained in the upper sieves and fluke eggs are retained in the bottom sieve. As most debris is removed by filtration, the subsequent sedimentation step is brief; however, such methods entail a risk of contamination by carryover. To minimize this risk, we used a method in which samples were only filtered through a sieve that removed larger faecal debris, and in which the sedimentation step was longer. In the case of the eMM3-COPRO, the risk of cross contamination was also minimized, as the plates were washed (the most critical point) with an automatic washer with a 96-tube manifold, i.e., each tube washes only a single well.

In summary, this prospective field study shows that the eMM3-COPRO test is a highly sensitive, specific and robust method for the diagnosis of sheep fasciolosis. As expected, it detects *Fasciola* infections both at late prepatent and patent stages and is superior to FEC for monitoring flukicide efficacy at different stages of the infection. In addition, the operational characteristics of this test make it particularly suitable for laboratories processing numerous samples.

## Limitations of the study

The diagnostic accuracy of the eMM3-COPRO (for a cut-off value of 0.021) proved better than that of coproscopy. However, we know that the eMM3-COPRO test can only detect coproantigens in sheep faeces from 5–7 weeks pi [11, 20]. In this context, earlier infections can only be accurately detected by necropsy or during processing in the slaughterhouse. However, it is essential to assess diagnostic tests in the target population where they are intended to be used, i.e. live animals in production. Antigen tests also cannot detect positive animals when the level of coproantigen is below the detection limit of the assay or when coproantigens are degraded during the intestinal transit. Consequently, we cannot totally rule out the possibility that a small number of samples from sheep with low concentrations of the *Fasciola* coproantigen (specifically those with OD values = 0.018–0.021) were erroneously classified as negative. Nevertheless, previous results from experimental infections in different ruminants seem to indicate that these rare cases are more frequent in cattle than in sheep [20]. By contrast, these limitations are not applicable to the specificity of the assay as no coproantigens were detected after TCBZ treatment for any positive sheep.

## Supporting information

S1 File (XLSX)
